# Supplementary material for: Prevalence of hypertension and its associated factors among government employees in Doti district of Nepal
Source: PLoS One. 2025 Aug 21;20(8):e0330753. doi: 10.1371/journal.pone.0330753 (PMC12370075; doi:10.1371/journal.pone.0330753)
Supplement: S1 Table — (DOCX) [file pone.0330753.s001.docx]

**S1 Table. Behavioral characteristics of the participants and its association with Hypertension**

**Tobacco consumption**

Among total respondents, about 4 in 10 were ever smoker of either smoked or smokeless tobacco products. Among ever smokers (n=81), 90.1% were current smokers and out of current smokers, 94.5% of the respondents were daily smokers of either smoked or smokeless tobacco products. The knowledge about tobacco product (control and regulate) act/regulation was low (43.1%) among participants and on observation, it was found that only one third (33%) of the Government offices among total study offices displayed information about tobacco control in respective offices as presented in Table 1.

| **S1A Table. Tobacco consumption related characteristics** | **(n=36)** | |
| --- | --- | --- |
| **Variables** | **Number** | **Percentage** |
| Ever smoking (n=195) | 81 | 41.5 |
| Current smoking (n=81) | 73 | 90.1 |
| Daily smoking (n=73) | 69 | 94.5 |
| Thought for quitting tobacco (n=73) | 37 | 50.7 |
| Knowledge on tobacco product(control and regulate) | 84 | 43.1 |
| act/regulation (n=195) |  |  |
| Displaying information to control tobacco in office | 12 | 33 |

Among current smokers (n=73), 17.8% of the respondents consumed smoked tobacco, 39.7% consumed smokeless tobacco, whereas, rest of the participants (42.5%) consumed both types of tobacco currently as presented in Table 2.

| **S1B Table. Types of tobacco (n=73)** | | |
| --- | --- | --- |
| **Current smoking** | **Number** | **Percentage** |
| Smoked tobacco | 13 | 17.8 |
| Smokeless tobacco | 29 | 39.7 |
| Both | 31 | 42.5 |

**Age at first smoking**

The mean age of initiation of smoking was 21.48 years with SD 6.1 years and the range included 11 years to 40 years. Among ever smokers, more than half of the participants (51.9%) started tobacco smoking within age group 10 to 20 years of age as presented in Table 3.

| **S1C Table. Age at initiation of smoking** | **(n=81)** |  |
| --- | --- | --- |
| **Age at first smoking** | **Number** | **Percentage** |
| Mean age = 21.48 years and SD = 6.1 years |  |  |
| 10-20 | 42 | 51.9 |
| 20-30 | 34 | 42.0 |
| 30-40 | 5 | 6.2 |
| **Frequency of tobacco consumption**  Among the participants who consumed smokeless | tobacco (n=60), | about one third |

(33.3%) of them consumed smokeless tobacco less than 5 times in a day, while 20% of them consumed more than 15 times per day. Among the participants who consumed smoked tobacco (n=44), more than half of the participants consumed smoked tobacco less than 5 sticks per day and about 16% of them consumed 10 to 15 sticks per day as presented in Table 4.

**S1D Table. Frequency of tobacco consumption per day**

| **Characteristics** | **Number** | **Percentage** |
| --- | --- | --- |
| **Smokeless tobacco (n=60)** |  |  |
| <5 times/day | 20 | 33.3 |
| 5-10 times/day | 17 | 28.3 |
| 10-15 times/day | 11 | 18.3 |
| >15 | 12 | 20.0 |
| **Smoked tobacco (n=44)** |  |  |
| <5 sticks/day | 25 | 56.8 |
| 5-10 sticks/day | 11 | 25.0 |
| 10-15 sticks/day | 7 | 15.9 |
| >15 sticks/day | 1 | 2.3 |

**Alcohol consumption**

Among the total participants, more than half (51.3%) of them had ever drunk alcohol in their lifetime and among them 87% of participants drank alcohol within 12 months. Nearly, three fourth (74.7%) of the participants consumed alcohol currently among those who drank alcohol within 12 months as presented in Table 5.

**S1E Table. Alcohol consumption related characteristics**

| **Variables** | **Number** | **Percentage** |
| --- | --- | --- |
| Ever drink alcohol (n=195) | 100 | 51.3 |
| Alcohol drink within 12 months (n=100) | 87 | 87 |
| Currently drinking alcohol (n=87) | 65 | 74.7 |
| Thought for quitting to drink (n=87) | 47 | 54 |

The mean age at first initiation of drinking alcohol was 22.41 years with SD 5.7 years and the range of 10 years to age 40 years. More than 40% among ever drunk participants initiated to drink alcohol within the age 10 to 20 years. More than two thirds (66.7%) of the participants drunk alcohol occasionally as presented in Table 6.

**S1F Table. Age at first drinking and frequency of alcohol consumption**

| **Variables** | **Number** | **Percentage** |
| --- | --- | --- |
| Mean age = 22.41 years, SD= 5.7 years |  |  |
| **Age at first drinking (n=100)** |  |  |
| 10-20 | 41 | 41.0 |
| 20-30 | 54 | 54.0 |
| 30-40 | 5 | 5.0 |
| **Frequency of alcohol consumption (n=87)** |  |  |
| Daily or almost daily | 11 | 12.6 |
| Weekly | 12 | 13.8 |
| Once a month | 6 | 6.9 |
| On occasion | 58 | 66.7 |

**Diet consumption**

Regarding dietary intake of fruit and vegetables, only 29.7% of total participants consumed sufficient fruits and vegetables and more than 45% of them consumed more amount of salt in a day than recommended amount as presented in Table 7.

| **S1G Table. Diet consumption** |  | **(n=195)** |
| --- | --- | --- |
| **Variables** | **Number** | **Percentage** |
| **Dietary intake (fruit and vegetables)** |  |  |
| Insufficient | 137 | 70.3 |
| Sufficient | 58 | 29.7 |
| **Salt intake** |  |  |
| Right amount intake ( ≤ 1 teaspoonful/day) | 75 | 54.7 |
| More amount intake (≥ 1 teaspoonful/day) | 62 | 45.3 |

**Physical activity**

Regarding physical activities, more than half (52.3%) of study participants had done any type of physical activities either vigorous, or moderate or insufficient. Only 18.6% of participants had done vigorous exercise as a part of physical activity and nearly half of them had done moderate physical activities as presented in Table 8.

| **S1H Table. Physical activity** |  | |
| --- | --- | --- |
| **Variables** | **Number** | **Percentage** |
| Doing physical activities (n=195) | 102 | 52.3 |
| Vigorous activities (n=102) | 19 | 18.6 |
| Moderate activities (n=83) | 41 | 49.4 |

Regarding the level of physical activities, only 18.6% of participants had done high level of physical activities and 40% of the participants had done insufficient level of physical activities as presented in Table 9.

| **S1I Table. Level of Physical activity among participants** |  | n=102 |
| --- | --- | --- |
| **Variables** | **Number** | **Percentage** |
| High (3000MET-minutes/week) | 19 | 18.6 |
| Moderate (600MET-minutes/week) | 41 | 40.2 |
| Insufficient (<150 minutes physical activity/week) | 42 | 41.2 |

**Blood pressure related information**

About three fourth (76.4%) of the total participants had ever measured their blood pressure from health workers and among them 30.2% had raised blood pressure. Among the participants who had raised blood pressure, 84.4% were being advised for taking antihypertensive medication but about 16% of them didn’t take medication. Among the participants who had ever taken antihypertensive medicine, 90.6% were currently on medication as presented in table 10.

**S1J Table. Blood pressure related information among participants**

| **Variables** | **Number** | **Percentage** |
| --- | --- | --- |
| Ever measured blood pressure (n=195) | 149 | 76.4 |
| Ever told for raised blood pressure (n=149) | 45 | 30.2 |
| Ever advised for taking medication (n=45) | 38 | 84.4 |
| Ever taken medicine (n=38) | 32 | 84.2 |
| Currently taking medicine (n= 32) | 29 | 90.6 |
| **Family history of blood pressure** |  |  |

The table below shows that about 39.5% of the participants had family history of hypertension and among them 36.4% are currently on antihypertensive medication as presented in table 11.

| **S1K Table. Family history of blood pressure** |  | n=195 |
| --- | --- | --- |
| **Variables** | **Number** | **Percentage** |
| Family member with hypertension | 77 | 39.5 |
| Family member on medication | 71 | 36.4 |

### Bivariate Analysis

### Association between tobacco consumption behaviour and hypertension

The table below shows the association between tobacco consumption behaviour of the participants with hypertension. The results show that ever smoking participants were significantly associated with hypertension (p-value<0.05, OR 2.17 (95% CI; 1.19, 3.93). Daily smokers were in 2.6 times more risk of developing hypertension than those with no daily smokers. The table shows that consuming more frequency of smokeless tobacco is significant associated with hypertension at p-value 0.035 (OR 5.57 with CI 95%; 1.12, 27.52) as presented in table 12.

### S1L Table. Association between tobacco consumption and hypertension

| **Variables** | **COR** | **CI (95%)** | **p-value** |
| --- | --- | --- | --- |
| **Ever smoking tobacco** |  |  |  |
| No | Ref |  |  |
| Yes | 2.17 | (1.19, 3.93) | .011* |
| **Age at first smoking** |  |  |  |
| 10-20 | Ref. |  |  |
| 20-30 | 0.87 | (0.35, 2.15) | .761 |
| 30-40 | 1.65 | (.25, 10.910) | .603 |
| **Current smoking** |  |  |  |
| No | Ref |  |  |
| Yes | 0.49 | (0.11, 2.23) | .359 |
| **Daily smoking** |  |  |  |
| No | Ref. |  |  |
| Yes | 2.6 | (0.26, 26.19) | .41 |
| **Types of tobacco** |  |  |  |
| Smoked tobacco | Ref. |  |  |
| Smokeless tobacco | 2.41 | (0.604, 9.27) | .213 |
| Both | 1.85 | (0.47, 7.32) | .379 |
| **Consumption of Smokeless tobacco (frequency)** | | | |
| Less than 5 | Ref. |  |  |
| 5-10 | 1.01 | (0.26, 3.92) | .985 |
| 10-15 | 3.25 | (0.701, 15.07) | .132 |
| More than 15 | 5.57 | (1.12, 27.52) | .035* |

### Association between alcohol consumption behavior and hypertension

The table below shows the association of alcohol consumption behavior of participants with hypertension. The study found that the ever drinker were found to be associated with hypertension which was statistically significant at 95% level with OR= 3.19 and CI is from 1.72 to 5.91. The participants who drank weekly were found to be statistically significant with hypertension at p-value= 0.027, OR 4.91 and CI from 1.19 to 20.11 as presented in table 13.

### S1M Table. Association between alcohol consumption and hypertension

| **Variables** | **COR** | **CI (95%)** | **p-value** |
| --- | --- | --- | --- |
| **Ever drinker**  No | Ref |  |  |
| Yes | 3.19 | (1.72, 5.91) | < .01* |
| **Age at first drinking** |  |  |  |
| 10-20  20-30 | Ref  1.08 | (0.48, 2.42) | .861 |
| 30-40 | 4.63 | (0.48, 45.09) | .187 |
| **Current Drinker** |  |  |  |
| No | Ref |  |  |
| Yes | 0.857 | (0.33, 2.26) | .755 |
| **Frequency of drinking** |  |  |  |
| Occasionally  Daily | Ref.  1.96 | (0.54, 7.21) | .309 |
| Weekly | 4.91 | (1.19, 20.11) | .027* |
| Once a month | 3.27 | (3.27, 19.37) | .191 |

* - statistically significant association with hypertension

### Association between dietary intake and hypertension

The table below shows the association of dietary behavior of participants with hypertension. Both the dietary intake (fruits and vegetables ≥5 servings daily) and salt intake were not found to be statistically significant as presented in table 14.

### S1N Table. Association of dietary behaviour and hypertension

| **Variables** | **COR** | **CI (95%)** | **p-value** |
| --- | --- | --- | --- |
| **Dietary intake (fruit and vegetables)** |  |  |  |
| Insufficient (< 5 servings/day) | Ref. |  |  |
| Sufficient (≥ 5 servings/day) | 0.78 | (0.32, 1.85) | .57 |
| **Salt intake** |  |  |  |
| Right amount (≤ 1 teaspoonful/day) | Ref. |  |  |
| More intake (> 1 teaspoonful/day) | 1.10 | (0.54, 2.23) | .79 |

###

### Association between physical activity and hypertension

The table shows the association of physical activity of participants with hypertension. It was found that the participants who did not have any physical activity had 1.41 times more risk of having hypertension than those who had done physical activity and also the participants who had moderate or insufficient physical activity had 1.12 times and 2.17 times more risk having hypertension as compared to those who had done high level of physical activity as presented in table 15.

### S1O Table. Association of physical activity with hypertension

| **Variables** | **COR** | **CI (95%)** | **p-value** |
| --- | --- | --- | --- |
| **Doing physical activity (n=195)** |  |  |  |
| Yes | Ref. |  |  |
| No | 1.41 | (0.78, 2.54) | .251 |
| **Level of physical activity (n=102)** |  |  |  |
| High | Ref. |  |  |
| Moderate | 1.12 | (0.35, 3.59) | .844 |
| Insufficient | 2.17 | (0.69, 6.78) | .184 |

### Association between family history of hypertension and hypertension

The participants with family history of hypertension was not statistically significant with hypertension at p-value>0.05 as presented in table 16.

### S1P Table. Association of family history with hypertension

| **Variables** | **COR** | **CI (95%)** | **p-value** |
| --- | --- | --- | --- |
| **Family member with hypertension**  No | Ref. |  |  |
| Yes | 0.91 | (0.49, 1.65) | .753 |
