# Supplementary material for: Prevalence of hypertension and its associated factors among government employees in Doti district of Nepal
Source: PLoS One. 2025 Aug 21;20(8):e0330753. doi: 10.1371/journal.pone.0330753 (PMC12370075; doi:10.1371/journal.pone.0330753)
Supplement: S1 File — (DOCX) [file pone.0330753.s002.docx]

**S1 File. Operational Definitions**

**Government employee**

An individual currently working (either permanent or temporary employee) either in civil service, health service or miscellaneous service at federal or provincial or local level Government offices were considered as Government employee.

**Socio-demographic variables**

These variables were related to age, sex, marital status, ethnicity, religion, family history of hypertension. Ethnicity was categorized according to Government of Nepal [10].

**Physical activity**

Physical activity was categorized into three groups; high, moderate and insufficient.

High: A person having vigorous and intensity activity on at least 3 days achieving a minimum of at least 1500 MET-minutes/ week or, ≥7 days of any combination of walking, moderate or vigorous intensity activities achieving a minimum of at least 3000 MET-minutes/week [25].

Moderate: A person having ≥3days of vigorous and intensity activity of at least 20 minutes/day or, ≥5 days of moderate-intensity activity or walking of at least 30 minutes/day or, ≥5 days of any combination of walking, moderate or vigorous intensity activities achieving a minimum of at least 600 MET-minutes/week [25].

Insufficient: A person having < 150 minutes of moderate or intensity physical activity

/week, or, equivalent [25].

**Diet**

*Consumption of fruit and vegetables:* Consumption of >5 serving of fruit and/or vegetables on average/day was considered as sufficient consumption and that of <5 serving of fruit and, or vegetables on average/day was considered as insufficient consumption [25].

One serving equivalent to 80 grams.

*Salt:* Dietary salt includes unrefined salt, iodized salt. Intake of up to 5 gram (equivalent to one teaspoonful) salt daily was considered as right amount of salt consumption while more than 5 gram salt consumption in daily diet was considered as more consumption of salt. A person who added salt at prepared meal and/or consumed salty processed food were considered as more consumption of salt. [25].

**Tobacco use**

*Smokeless tobacco:* Chewing tobacco, snuff, betel, tamakhu, gutkha was considered as smokeless tobacco.

*Smoke tobacco:* Cigarettes, cigar, pipes, hukka was considered as smoking.

*Tobacco use:* A person was considered as current tobacco user if he/she used tobacco (smoking or smoke less) in the last 30 days [25].

**Alcohol use**

*Never:* A person who never drank alcohol.

*Stop drink:* A person who didn’t drink alcohol during the past 12 months

*Current user:* A person who drank alcohol at least once during the past 30 days [25].
