# Supplementary material for: Prevalence of hypertension and its associated factors among government employees in Doti district of Nepal
Source: PLoS One. 2025 Aug 21;20(8):e0330753. doi: 10.1371/journal.pone.0330753 (PMC12370075; doi:10.1371/journal.pone.0330753)
Supplement: Attachment — Submitted filename: Response to Reviewers.docx [file pone.0330753.s003.docx]

Dear Chhabi Lal Ranabhat,

Academic Editor

PLOS ONE

I am writing in response to the comments provided by the reviewers regarding our manuscript PONE-D-25-05146 entitled **"Prevalence of Hypertension and its Associated Factors among Government Employees in Far Western Province of Nepal"**. We appreciate the time and effort invested by the reviewers and the editorial team in evaluating our work. After careful consideration of the reviewers' comments, we have addressed each point raised and provided our responses below:

**Reviewer #1**

1. **Comment**: *Title suggests that the study was conducted across the entire Far Western Province, but the data was only collected from Doti district. Please adjust the title to reflect the correct geographic scope.*

**Response**: We agree with the reviewer and have revised the title to **"Prevalence of Hypertension and its Associated Factors among Government Employees in Doti District of Nepal"** to accurately reflect the study's scope. (Line number 3)

2. **Comment**: *The adjusted odds ratio (AOR) for the 50–60 years age group (AOR 11.6, 95% CI 0.73, 6.5) is not statistically significant. Please revise the results and discussion accordingly.*

**Response**: We have revised both the results and the discussion sections to clarify that this finding is not statistically significant and should not be misinterpreted. (line number 40,41)

3. **Comment**: *Please add "Nepal" as a keyword.*

**Response**: We have added "Nepal" to the list of keywords. (line number 49)

4. **Comment**: *Specify exact data collection start and end dates (date/month/year).*

**Response**: We have updated the specific data collection dates in the revised manuscript **as 5th December 2022 to 4th January 2023**(line number 125)

5. **Comment**: *Please provide the response rate and add it to the limitations if necessary.*

**Response**: We have added the response rate (93%) to the methodology section. Since not all approached participants agreed to participate, we have also noted this as a limitation. (line number 130,289, 290)

6. **Comment**: *Consider merging variables in the regression analysis for clearer interpretation, such as grouping age categories into <40 and ≥40 years.*

**Response**: We appreciate this suggestion and we agree that merging age categories can sometimes help with clearer interpretation. However, during our regression analysis, we observed that the age groups 30–40 and 40–50 years showed significant associations with hypertension, while the 50–60 years age group did not show a statistically significant association. Therefore, we retained the current categorization to preserve these important distinctions in age-specific risk, which could be masked if broader categories (e.g., <40 and ≥40 years) were used.

7. **Comment**: *Remove the asterisk (*) for non-significant p-values in Table 3 and Table 4.*

**Response**: The asterisk (*) has been removed from non-significant p-values, and a footnote has been added to explain that p-values <0.05 are marked with an asterisk.

8. **Comment**: *Please add the p-values from bivariate analysis (COR) alongside those from the multivariate analysis (AOR) in Table 3 and Table 4.*

**Response**: We have included the p-values from both bivariate and multivariate analyses in the revised tables for clarity.

9. **Comment**: *Correct the interpretation of non-significant results, such as male being a significant risk factor for hypertension.*

**Response**: We have revised the discussion to accurately describe that being male was not a statistically significant risk factor for hypertension.

10. **Comment**: *Please add recommendations in the conclusion section.*

**Response**: We have added specific recommendations, including lifestyle interventions and workplace health programs, to address the high prevalence of hypertension among government employees. (line number 299-305)

11. **Comment**: *Please follow proper referencing style.*

**Response**: We have doubled checked and refined all the references to ensure to follow the appropriate referencing style as per PLOS ONE authors guidelines.

**Reviewer #2**

1. **Comment**: *Justify why the sample size was taken from Doti instead of the national proportion. Also, analyze the corrected margin of error in the results section.*

**Response**: Thank you for your valuable comment. The sample size of **195** was determined using **Cochran’s formula for finite population correction**, which is appropriate when the target population is relatively small and well-defined.

Although the **expected prevalence (p = 0.20)** of hypertension was taken from the **National Demographic and Health Survey (NDHS) of Nepal**, the **study population was limited to government employees in Doti district**. Therefore, it was both methodologically sound and necessary to use the finite population correction (FPC) based on the actual population of interest — i.e., the **total number of government employees in Doti (N = 625)**.

**Margin of Error Clarification:**

By applying the finite population correction, the **effective margin of error is slightly reduced** compared to the initial 5% assumption, leading to more precise estimates.

2. **Comment**: *Review the limitations of the Hosmer-Lemeshow test and interpret large confidence intervals for AOR (age group). Consider including additional model fit parameters, such as AIC or BIC.*

**Response**: Thankyou for your thoughtful comments. We acknowledge and have addressed the concerns regarding model fit evaluation and the interpretation of regression outputs.

**Limitations of the Hosmer-Lemeshow Test:** While applying the multivariate analysis, the Hosmer and Lemeshow test was performed to test the goodness of fit. The test result (p = 0.345) indicated good model fit. However, we recognize that the Hosmer-Lemeshow test has limitations (line number 291-294). It is **sensitive to sample size** and can yield misleading results in small samples like ours (n = 195), where it may fail to detect misfit. Furthermore, the grouping of predicted probabilities into deciles is arbitrary and may affect the outcome. Therefore, we agree that relying solely on this test may not provide a full picture of model adequacy.

**Interpretation of Wide Confidence Intervals (AORs):** As observed in Table 3, some adjusted odds ratios (AORs), particularly for **age groups 30–40 (AOR: 14.4; 95% CI: 1.6–127.7)** and **40–50 (AOR: 13.7; 95% CI: 1.04–180.3)**, have **very wide confidence intervals**, suggesting a high degree of uncertainty around the estimates. This is likely due to **small subgroup sizes or sparse data**, which can inflate standard errors and widen confidence intervals. Despite statistical significance, these estimates should be interpreted with caution, as the precision is limited. This limitation has now been explicitly mentioned in the **discussion section**. (line number 243-246)

3. **Comment**: *Correct the misinterpretation of non-significant results for factors such as male gender and physical activity (lines 239 and 254).*

**Response**: We have revised these sections to reflect that the male gender and physical activity were not statistically significant risk factors.

4. **Comment**: *Compare the significantly different hypertension prevalence rates (38% and 66%) between government employees in Nepal and other countries.*

**Response**: We have clarified the comparison between the different prevalence rates and provided additional context regarding the behavioral and lifestyle differences that might account for this disparity.(line number 231-234)

We believe that these revisions have addressed the concerns of both reviewers that helped to improve quality of our paper. We appreciate your valuable feedback and hope that the revised manuscript is now suitable for publication.

Thank you for your time and consideration.

Sincerely,
Sunita Sharma

On behalf of the co-authors
