# Supplementary material for: Prevalence of hypertension and its associated factors among government employees in Doti district of Nepal
Source: PLoS One. 2025 Aug 21;20(8):e0330753. doi: 10.1371/journal.pone.0330753 (PMC12370075; doi:10.1371/journal.pone.0330753)
Supplement: Attachment — Submitted filename: Recommendation.docx [file pone.0330753.s004.docx]

**Abstract:** line no 7, "which are the leading causes" could be revised into" which remains the leading causes of morbidity and mortality" (or you can revise on your own preferences)

In the methodology sections of the abstract, please add "structured questionnaire adopted from the WHO STEPS survey tool was used" would improve clarity for the reader.

**Introduction**

ln line 52 , Author has started with Cardiovascular disease (CVD) , it would be more effective to begin with hypertension itself, since it is the central focus of the study and then connect it to its role as a leading modifiable risk factor for CVDs.

Please include a brief rationale at the end of the introduction explaining why government employees in a rural district like Doti were selected as the study population.

**Methodology**
In line no 93 , You have added the term " Miscellaneous service " please specify what is meant by miscellaneous service ?

Lines 94–95: You have used "were not included." Please revise this to: "Currently employed government staff who were present during the data collection period were included in the study, while those who were on leave or absent were excluded."

Line 134 mentions the Omron automatic BP monitor, but line 141 refers to the random zero sphygmomanometer. These are two different instruments. Please clarify which was actually used.

**Results**

Line 181: Replace "+_SD" with "± SD."

In the table, please correct the spelling of “Brahmin.”

It is recommended to round the percentages in the education and service type categories so they sum exactly to 100%, for clarity and consistency.

Line 211: The finding that alcohol consumption appears to be a protective factor against hypertension (AOR < 1) is unusual and not supported by most studies. This may be due to a small number of drinkers in the study or information bias. It is recommended that the authors clearly interpret this result in the results section and acknowledge it as a potential limitation in the discussion.

**Discussion**

In the discussion section, it is suggested to restructure the comparison flow. The authors currently begin by comparing their findings with global and LMIC data, followed by Nepal-specific studies. Please revise the discussion flow to start with Nepalese studies, then South Asian/regional, and finally global data for better context.

Please add a reference to support the conclusion in lines 277–281 regarding interventions and prevention.

**Limitation:**

Please paraphrase lines 289 and 290 for better readability and flow.
