# Supplementary material for: Prevalence of hypertension and its associated factors among government employees in Doti district of Nepal
Source: PLoS One. 2025 Aug 21;20(8):e0330753. doi: 10.1371/journal.pone.0330753 (PMC12370075; doi:10.1371/journal.pone.0330753)
Supplement: Attachment — Submitted filename: Response_to_Reviewers_auresp_2.docx [file pone.0330753.s005.docx]

Respected Chhabi Lal Ranabhat,

Academic Editor

PLOS ONE

I am writing in response to the comments provided by the reviewers regarding our manuscript PONE-D-25-05146R1 entitled **"Prevalence of hypertension and its associated factors among government employees in Doti district of Nepal"**. We appreciate the time and effort invested by the reviewers and the editorial team in evaluating our work. After careful consideration of the reviewers' comments, we have addressed each point raised and provided our responses below:

### ****Reviewer 1****

**Abstract**
**Comment:** Line 7, “which are the leading causes,” could be revised.
**Response:** Revised to: “which remain the leading causes of morbidity and mortality globally.” [page no 2, line no26]

**Comment:** Methodology – Add clarity about the tool used.
**Response:** Added the sentence: “The structured questionnaire adopted from the WHO STEPS survey tool was used for data collection.” [page no 2, line no 35-36]

**Introduction**
**Comment:** Start with hypertension, not CVD, in line 52.
**Response:** Revised the paragraph to begin with hypertension and subsequently linked it to CVD as a modifiable risk factor as below. *[page no 3, line no 53-60]*

*Hypertension is a leading modifiable risk factor for cardiovascular diseases (CVDs) and premature mortality worldwide. Between 1990 and 2019, global deaths from CVDs increased steadily from 12.1 million to 18.6 million respectively [1]. The prevalence of hypertension among adults is higher in Low Middle Income Countries (LMICs) (31.5%, 1.04 billion people) than in High-Income Countries (28.5%, 349 million people) [2]. The rising prevalence of hypertension, coupled with low medication adherence, poor blood pressure control, and limited knowledge about the condition, presents significant challenges, particularly in low- and middle-income countries (LMICs) [2].*

**Comment:** Add rationale for selecting government employees in rural Doti.
**Response:** Included rationale at the end of the introduction as below. *[page no 3, line no 84-88]*

*Doti district, one of the underprivileged rural and hilly areas of Nepal has insufficient health data, especially when it comes to non-communicable diseases like hypertension. Studying prevalence of hypertension among government employees in a rural setting like Doti district not only closes a significant evidence gap but also contributes to the development of targeted strategies for the prevention and management of hypertension in underprivileged areas of Nepal.*

**Methodology**
**Comment:** Line 93 – Clarify "Miscellaneous service."
**Response:** Revised to specify: “*Miscellaneous service includes other gazetted-level government employees falling outside administration or health like audit, foreign affairs, statistics, engineering, agricultural, and other services*” [page no 4, line no 102-104]

**Comment:** Lines 94–95 – Revise inclusion/exclusion phrasing.
**Response:** Revised to: “Currently employed government staff who were present during the data collection period were included in the study, while those who were on leave or absent were excluded.” [page no 4, line no 104-106]

**Comment:** Clarify discrepancy between Omron monitor and random zero sphygmomanometer.
**Response:** Clarified in the manuscript: “Blood pressure was measured using an Omron automatic BP monitor and no random zero sphygmomanometer was used” [page no 6, line no 146-147]

**Results**
**Comment:** Line 181 – Replace "+_SD" with "± SD."
**Response:** Corrected to “± SD.” [page no 7, line no 193]

**Comment:** Correct spelling of “Brahmin” in the table.
**Response:** Spelling has been corrected. [page no 8, line no 200]

**Comment:** Round percentages to sum to 100%.
**Response:** Percentages have been rounded appropriately to ensure they sum to 100% in the education and service-type categories. [page no 8, line no 200]

**Comment:** Alcohol consumption appears protective; interpret and note as limitation.
**Response:** We have noted the unusual finding, and acknowledged it in the discussion as a potential limitation as, [page no 12, line no 263-269] and in the limitations [page no 14, line no 324-326]

*Interestingly, alcohol consumption in our study showed an inverse association (AOR :0.35; 95% CI: 0.17-0.72), potentially reflecting reverse causation, reporting bias, or unmeasured confounding. It is possible that this observed association is influenced by residual confounding or reverse causation, whereby individuals with existing health conditions may reduce or abstain from alcohol use. Moreover, potential underreporting or misclassification of alcohol intake cannot be ruled out. This is a notable limitation of the study and underscores the need for longitudinal research to further explore this relationship.*

**Discussion**
**Comment:** Restructure comparison flow from global to local.
**Response:** Revised the discussion structure flow from global to local context. [page no 11-13, line no 238-296]

**Comment:** Add reference to support intervention/prevention statements (lines 277–281).
**Response:** A supportive interventions are added and proper reference has been added *as below [page no 13, line no 296].*

*Interventions tailored to workplace environments such as encompassing routine screenings, healthy diet and physical activity promotion, and alcohol education [22] are critically needed to address this emerging public health challenge.*

**Limitation**
**Comment:** Paraphrase lines 289–290 for better clarity.
**Response:** Revised to: “*The study adopted a cross-sectional design, which may preclude the establishment of causality with the identified risk factors.* [page no 13 , line no 299-300]

### ****Reviewer 2****

**Comment 1:** Re-analysis with corrected margin of error for precision.
**Response:** *We acknowledge the oversight. The margin of error has been corrected in the statistical analysis. The updated confidence intervals and interpretations are reflected in the results section and aligned appropriately in the discussion as: (page no 8-9, line no 201-210)*

*The prevalence of hypertension among government employee was found to be 36.4% ± 5.6% (36.4% ± 5.6%) (95% confidence, with finite population correction) as presented in Table 2. The margin of error for sample size (n=195) and total number of government employees in Doti (N = 625) with 95% Confidence Interval has been calculated as follows,*

**Step 1: Standard Error (without FPC)**

$SE= \sqrt{\frac{p(1-p)}{n}}= \sqrt{\frac{0.364(1-0.364)}{195}}= \sqrt{0.0011872}\approx0.03447$ $\boldsymbol{Step 2: Finite Population Correction}\left( \boldsymbol{FPC} \right)$

$$\boldsymbol{FPC=}\sqrt{\frac{N-n}{N-1}}= \sqrt{\frac{625-195}{624}}= \sqrt{\frac{430}{624}}= \sqrt{\left\{ 0.688 \right\}}\approx0.8294$$

**Step 3: Corrected Margin of Error (MoE)**

$$MoE=z\times SE\times FPC=1.96\times0.03447\times0.8294=1.96\times0.02859\approx0.05604$$

Margin of Error= 5.6%

**Prevalence of hypertension=36.4% ± 5.60% (95% confidence, with finite population correction)**

**Comment 2:** Clarify fitness of model in absence of H-L test, AIC, or BIC.
**Response:** We appreciate the reviewer’s valuable suggestion regarding additional metrics for model fit assessment. In our analysis, we used the **Hosmer–Lemeshow (H–L) test** to evaluate the goodness of fit for both models. The first model yielded a p-value of **0.345**, and the second model had a p-value of **0.820**—both exceeding the conventional threshold of 0.05, indicating acceptable model fit and no significant difference between observed and predicted probabilities.

In addition, we reported the **Nagelkerke R²** values, which were **0.445 and** **0.091**, respectively. These values suggest that the explanatory variables in the first model accounted for approximately 45% of the variation in the dependent variable, which represents a moderate explanatory power in epidemiological modeling. While the R² for the second model was relatively low, such values are typical in cross-sectional studies where numerous unmeasured or behavioral factors may influence outcomes.

Regarding AIC or BIC, we acknowledge their usefulness in model comparison and selection. However, as our analysis platform did not provide log-likelihood outputs, calculation of AIC/BIC was not feasible. But we believe that the combination of the Hosmer–Lemeshow test and Nagelkerke R² provides a standard and acceptable evaluation of model fit in cross-sectional logistic regression, particularly within resource-constrained research settings. Further, we have also noted the limitations of these methods in the revised manuscript.

We believe that these revisions have satisfactorily addressed the reviewers' comments and significantly improved the quality of our manuscript. We sincerely appreciate your valuable feedback and hope that the revised manuscript meets the publication standard of your reputed journal. Thank you for your time and consideration.

Sincerely,
Sunita Sharma

On behalf of the co-authors
