# Supplementary material for: Prevalence of hypertension and its associated factors among government employees in Doti district of Nepal
Source: PLoS One. 2025 Aug 21;20(8):e0330753. doi: 10.1371/journal.pone.0330753 (PMC12370075; doi:10.1371/journal.pone.0330753)
Supplement: Attachment — Submitted filename: comment.docx [file pone.0330753.s006.docx]

- Line 49: Keywords should be arranged in alphabetical order and avoid abbreviations like “CVD”; use full terms such as cardiovascular disease instead.
- Line 65: Consider replacing the word “patients” with “individuals”, as the context refers to population-level data from a meta-analysis rather than clinical patients.
- Kindly clarify whether the assumptions of logistic regression were checked—such as multicollinearity, model fit (e.g., using the Hosmer-Lemeshow test), and goodness-of-fit statistics. Also, specify the type of regression model used and clearly describe the criteria for selecting variables into the multivariate model.
- In the discussion section, please clarify the statement about the range of hypertension prevalence. A more accurate phrasing would be: “The prevalence of hypertension among government employees in this study was found to be 36.4% (95% CI: 30.8%–42.0%).”
